# Reduction Mammoplasty: Intraoperative Weight Versus Pathology Weight and Its Implications

**Published:** 2017-10-12

**Authors:** Matthew R. Zeiderman, Shahrooz Sean Kelishadi, John Paul Tutela, Saeed Chowdhry, Ronald M. Brooks, Bradon J. Wilhelmi

**Affiliations:** Division of Plastic and Reconstructive Surgery, Hiram C. Polk Jr. M.D. Department of Surgery, University of Louisville School of Medicine, Louisville, Ky.

**Keywords:** reduction mammoplasty, breast, reduction, weight, specimen, insurance, third party, reimbursement, implications, compensation

## Abstract

**Background:** Despite the efficacy of reduction mammoplasty and demonstration that resection weight does not predict symptomatic relief of macromastia, many insurers still rely on the Schnur scale or predetermined resection weight for reimbursement. Insurers review pathology reports to determine reimbursement. Tissue desiccation and handling decrease specimen weight prior to pathology evaluation. Surgeons often make judgments based on intraoperative weight. Our goal was to determine whether (1) discrepancies exist between intraoperative and pathology weights, and (2) how differences may impact reimbursement and medical practice. **Methods:** Medical records review was performed on 25 reduction mammoplasty cases performed between 2007 and 2010, yielding 48 specimens. Tumescent was never used. Weight of each specimen from operative and pathology reports was reviewed and compared. The 2-sample Kolmogorov-Smirnov test was used to compare sample weights. **Results:** Comparison of intraoperative versus pathology specimen weights revealed an average 7% weight decrease (range, +11% to −45%). Average and median specimen weight decrease from intraoperative to pathology weights was 48 g (SD = 71 g) and 31 g (interquartile range = 6.6-58 g), respectively. Average intraoperative specimen weight was 780.7 g (SD = 375.3 g; range, 290-2238 g). Average pathology specimen weight was 732.3 g (SD = 358.4 g; range, 265-2053.6 g) (*P* < .001) All but 2 samples weighed less in pathology. **Conclusion:** Desiccation and handling between intraoperative and pathology weighing decrease specimen weight. Weight discrepancies may have implications on coverage and reimbursement by insurers. Awareness of such discrepancies can help plastic surgeons and patients avoid unexpected coverage and reimbursement complications.

Reduction mammoplasty (RM) is a commonly performed surgical procedure to enhance a woman's quality of life, relieve symptoms of macromastia, and improve the aesthetics of large ptotic breasts. According to the American Society of Plastic Surgeons, 100,850 RMs were performed for women in 2015.^1^ Of these, 60,175 qualified as reconstructive procedures, making it the seventh most commonly performed reconstructive case. RM is indicated for the relief of physical symptoms that include pain and soreness of the back, hips, neck, shoulders, and also painful bra strap pain. Numerous studies have demonstrated this procedure's effectiveness at alleviating both somatic and psychological symptoms of macromastia,^2^^-^8 giving support to have insurance companies classify RM as a reconstructive procedure.

Many insurance companies have set criteria for RM reimbursement. Requirements vary by insurer but commonly include age greater than 18 years, attempts at medical management, and a predetermined resection weight of breast tissue of more than 500 g.^9^^-^11 The breast tissue resection weight requirement is particularly challenging. Smaller women with symptoms of macromastia may not meet this predetermined resection weight threshold and be denied coverage. For others, removal of an insufficient mass of breast tissue may result in a denial of payment to the surgeon by the insurance company or the burden of payment unexpectedly falls upon the patient. These issues are problematic within the plastic surgery community, as multiple studies have demonstrated that resection weight does not correlate with symptomatic relief of macromastia.^2^^,^^12^^-^14 Despite these studies, the third-party payer environment in which many plastic surgeons practice necessitates meeting RM reimbursement requirements and diligent preapproval to ensure compensation for patient service. Further complicating the compensation issue is that the weight measured by pathologists and recorded is frequently less than that measured intraoperatively by the surgeon. This is due to fluid loss upon removal from the body, tissue desiccation from air exposure, and handling and contact with desiccants such as towels.^15^^-^17 This is problematic because the weight in the pathology report can be reviewed by third-party payers prior to physician reimbursement. In an effort to help plastic surgeons better understand the impact of discrepancies in resection weight measurements used by the surgeon compared with that used by third-party payers, we present a retrospective analysis of 25 RM procedures comparing intraoperative with pathology laboratory resection weights.

## METHODS

University of Louisville School of Medicine institutional review board approval was attained prior to commencement of this retrospective study. Medical records review was performed on 25 RM cases performed between 2007 and 2010, which yielded 48 specimens. Twenty-three cases were bilateral RM and 2 were unilateral RM. All cases were either traditional Wise pattern inferior pedicle reduction or vertical RM based on the technique described by Hall-Findlay.^18^^,^^19^ All operations were performed under the supervision of the senior author (B.J.W.). Tumescent was not used for any reductions. Recorded weight of each specimen from the operative report and pathology report was reviewed and compared on all 48 specimens. Intraoperative weights were recorded immediately after specimen collection. Pathology weights were ascertained from the patient's pathology report. Only RM cases with both intraoperative and pathology weights available in the patient chart were included. The 2-sample Kolmogorov-Smirnov (KS) test was used to compare intraoperative and pathology weights. All cases received third-party preauthorization prior to operation.

## RESULTS

Average age for the patients analyzed in this study was 42 years (range, 25-68 years). Average intraoperative specimen weight was 780.7 g (SD = 375.3 g; range, 290-2185 g). Based on intraoperative weights, 8 of 48 specimens (17%) were less than 500 g, 16 of 48 specimens (33%) were between 501 and 750 g, 17 of 48 specimens (35%) were between 751 and 1000 g, and 7 specimens (15%) were greater than 1000 g. Pathology weights recorded for 10 specimens (21%) as less than 500 g, 17 of 48 specimens (35%) as between 501 and 750 g, 14 of 48 specimens (29%) between 751 and 1000 g, and 7 of 48 specimens (15%) as greater than 1000 g (Table 1).

**Figure 1:**
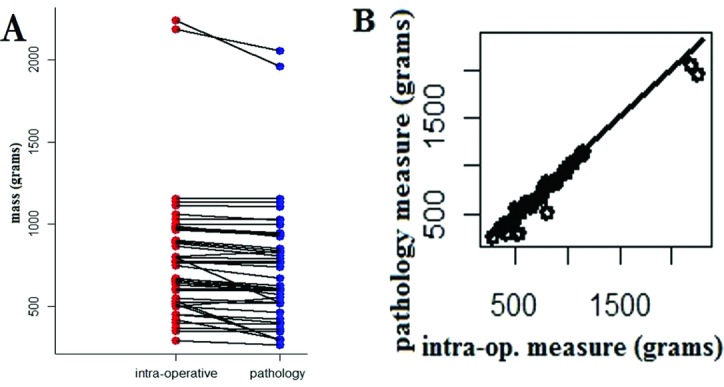
(*a*) Graphical representation of discrepancies between intraoperative and pathology laboratory recorded weights; (*b*) graphical representation and statistical analysis with the 2-sample Kolmogorov-Smirnov test of the 2-sample populations (intraoperative vs pathology weight) demonstrated statistically significant difference between the 2 data distributions (*P* < .001).

Comparison of intraoperative versus pathology laboratory specimen weights revealed an average 7% decrease in weight (range, +11% to −45% change in weight). Average and median specimen weight decrease from intraoperative to pathology laboratory weights was 48 g (SD = 71 g) and 31 g (interquartile range [IQR] = 6.6-58), respectively. Average intraoperative specimen weight was 780.7 g (SD = 375.3 g; range, 290-2238 g). Average pathology specimen weight was 732.3 g (SD = 358.4 g; range, 265-2053.6 g). All but 2 samples were recorded to weigh less in the pathology laboratory than the intraoperative weight. Graphical representation is available in Table 2 and Figure 1*a*.

Statistical analysis with 2-sample KS test of the 2-sample populations (intraoperative vs pathology weight) demonstrated statistically significant difference between the 2 data distributions (*P* < .001) (Fig 1*b*).

No breasts required reoperation. Minor postoperative complications included 3 breasts with evidence of fat necrosis that resolved with conservative management and 2 breasts with loss of nipple sensation.

Comparison of University of Louisville Hospital RM reimbursement rates found 6 of 8 insurers to require a minimum resection weight of 750 g per specimen. Two companies utilized an insurance scale for establish coverage criteria (Table 3).

## DISCUSSION

A 2002 study by Kaplan et al^20^ compared intraoperative with pathology laboratory breast RM resection weights of specimens when using the tumescent technique versus traditional inferior pedicle RM. The study showed that 9% of all breasts using the tumescent technique and 13% of breasts resected with a traditional RM technique were above required insurance estimates in the operating room but fell below the estimated insurance compensation requirement upon measurement in the pathology laboratory.^20^ That study reported a median decrease between the average weight of breast specimens of 2.0 g (IQR = −19.5 to +15.0 g) in the traditional inferior pedicle RM group and 67.3 g (IQR = −91.0 to −30.7 g) decrease for reductions utilizing the tumescent technique.^20^ The greater decrease in specimen weight found in the tumescent group may be explained by the seepage of unspecified volumes of tumescent used for the procedure, although it is not possible to ascertain how much weight tumescent contributed to those samples.

Our retrospective study using traditional RM techniques without the use of tumescent infiltration finds an average 48-g decrease in specimen weight upon pathology laboratory measurement, with a median decrease of 31 g (IQR = 6.6-58 g). Our study shows a significant decrease in average specimen weight due to tissue desiccation from to air exposure, tissue handling, and exposure to desiccants. Our findings highlight the importance of prompt weighing of RM specimens in the pathology laboratory following surgical resection, as tissue handling and desiccation quickly decrease the weight of the specimens. Such decreases in weight may have a negative impact on third-party payer reimbursement if the insurer utilizes a predefined resection weight or scaled weight based on body mass index or body surface area (BSA).

Historically, many third-party payers have based the required resection weight on the Schnur scale.^10^^,^^21^ The Schnur scale is an algorithm developed from a survey of the opinion of plastic surgeons to determine weight resection requirements for symptomatic relief of macromastia based on BSA.^10^^,^^21^ According to this scale, RMs for patients above the 22nd percentile are deemed medically necessary whereas those below the 5th percentile are classified as cosmetic. This leaves a significant gray area for preauthorization disputes with insurers for the patient whose insurer utilizes the Schnur scale for determining eligibility criteria. However, the Schnur scale was never intended to serve as a guideline for insurance coverage criteria, as recognized by Dr Schnur himself.^10^^,^^22^ Despite this, a study by Frey and colleagues^9^ using a representative survey of American insurance companies found 12 of 15 utilize the Schnur scale to determine weight resection requirements and 14 of 15 (except Medicare) have minimum resection weight requirements. A larger survey study of 90 insurance companies from across the country performed by Nguyen and colleagues^10^ in 2008 found that 51 of 90 use the Schnur scale to determine resection criteria and 40 of 90 require a minimum resection weight independent of height and weight, with an average resection requirement of 426 g per specimen. Overall, the Nguyen and colleagues study finds medical policy requirements for RM of the sampled third-party payers to be largely nonuniform, somewhat arbitrary, and unsupported by scientific data. The only other peer-reviewed study to attempt to correlate resection weight with medical necessity was published in 1995 by Seitchik,^23^ who attempted to plot weight of tissue resection compared with patient body weight. The author was unable to derive a useful formula to determine medical necessity based on the data associations but proceeded to personally recommend a formula for reduction weight based upon body weight without utilizing the study data. This recommended formula entails 3 levels of recommended minimum resection weight based upon patient body weight. Like the Schnur scale, using this method to determine medical necessity based upon a single surgeon's experience is also flawed, as it fails to appropriately correlate resection weight with medical necessity.

Others have tried to develop a formula or method for preoperative prediction of breast resection weights. These are based on anatomic measurements such as sternal notch to nipple, sternal notch to inframammary crease, and nipple to inframammary crease,^21^^,^^24^^,^^25^ bra and cup size measurements,^26^^,^^27^ and mass of the ptotic breast.^28^ However, these methods fail to consistently and accurately predict true resection weight and volume.^21^^,^^24^^,^^29^ Prediction weights have some utility as an aid to the plastic surgeon when estimating resection weight; however, resection of the appropriate amount of breast tissue to produce an efficacious, aesthetically pleasing result ultimately relies on the surgeon's intraoperative judgment. What is perplexing about the current reimbursement environment is that multiple studies have shown that predetermined resection weight and volume do not correlate with symptom alleviation and therefore should not be used to determine medical necessity.^2^^,^^12^^,^^14^^,^^30^^,^^31^ Two studies by Spector and colleagues^2^^,^^12^ demonstrate that women with a broad spectrum of breast size experience similar preoperative symptoms, yet subsequent postoperative symptom relief is similar among these women independent of resection volume. Recently, a 2015 study by Strong and colleagues^13^ finds a statistically significant postoperative improvement in all symptoms for breast volumes ranging from less than 251 g to more than 550 g, including those who would be considered for cosmetic procedures under many insurance reimbursement criteria such as the Schnur scale or predetermined minimal resection weight. Despite these findings, RM insurance criteria remain unchanged.

For larger RMs, the discrepancy between intraoperative and pathology laboratory weights is of less significance. However, for cases in which smaller tissue resection mass is necessary to alleviate symptoms and produce an aesthetic result, this weight discrepancy may determine whether or not the plastic surgeon will be compensated for his or her work. The issue of compensation for service may be avoided by meticulous efforts for preauthorization with third-party payers, but despite these efforts, insurers may still perform case reviews and deny reimbursement. Our study found that RM specimens decrease an average of 48 g upon weighing in the pathology laboratory. This is a significant amount of weight. For plastic surgeons who rely on minimal resection requirements for RM reimbursement from insurers, this is particularly problematic. This situation may be even more troubling for the patients who are unexpectedly charged a higher bill than anticipated, which they might not be prepared or able to pay. With this is mind, plastic surgeons must be diligent about keeping in mind the need for preapproval or seek institutional policies that ensure prompt weighing of RM specimens. For plastic surgeons employed by universities or hospitals, this may be pertinent to the employer's perspective as well. Others in private practice may need to be even more industrious, as delays in transfer from the operating room to the pathology laboratory could adversely impact individual surgeon's compensation and patient's health care expenses.

Our study is not without limitations. It is possible that some specimens spent significantly more time in storage or transport between documentation of intraoperative weights and weighing in the pathology laboratory. Despite our diligence, scales may have been inappropriately zeroed or specimens weighed inaccurately. Inaccurate weights may have been recorded in the medical record, or staff errors in recording of specimen weight may have occurred. Finally, the retrospective nature of this study might have prevented us from attaining the records of other patients who underwent RM within the defined period. Despite the possible limitations of our study, we hope these findings will help better protect our colleagues against getting insurance claims denied for the work required in performing RM.

## CONCLUSIONS

In this study, we find that RM breast tissue specimen desiccation and handling between intraoperative and pathology laboratory weight recordings result in an average of 7%, or 48 g, decrease in specimen weight. These findings were observed without the use of tumescent, which can add indeterminate weight to resected tissue. Given that insurance company reimbursement criteria often utilize the Schnur scale or a predetermined resection weight, these differences in recorded weight between intraoperative and pathology laboratory weighing may have profound implications. We hope the findings of this study will enrich the plastic surgery literature and that plastic surgeons will consider these implications in their practice setting.

## Figures and Tables

**Table 1:** Specimen weight categorization^*^

Specimen weight, g	Intraoperative specimens	Pathology specimens
<500	8 (17%)	10 (21%)
501-750	16 (33%)	17 (35%)
751-1000	17 (35%)	14 (29%)
>1000	7 (15%)	7 (15%)

*Comparison of weight range classification for specimens between intraoperative and pathology laboratory specimens.

**Table 2:** Reduction mammoplasty recorded weights and discrepancies^*^

	Intraoperative weight, g	Pathology weight, g	% Decrease	Actual decrease, g
	966	934	3	32
	600	595	1	5
	660	605	8	55
	511	296	42	215
	882	824	7	58
	370	359	3	11
	977	946	3	31
	670	606	10	64
	607.7	595	2	13
	900	852	5	48
	865	807	7	58
	500	462	8	38
	2185	2053.6	6	131
	450	424	6	26
	893	840	6	53
	630	600	5	30
	1154	1154	0	0
	290	265	9	25
	606	605	0	1
	450	401.2	11	49
	801	834	+4	+33
	1030	1030	0	0
	764	760	1	4
	776	740	5	36
	610	595	2	15
	645	606	6	39
	530	292	45	238
	796	790	1	6
	400	393.2	2	7
	1061	1024	3	37
	665	626	6	39
	649.1	570	12	79
	1000	995.7	0	4
	864	805	7	59
	525	518	1	7
	2238	1960	12	278
	750	670	11	80
	550	521	5	29
	986	925	6	61
	500	556	+11	+56
	1115.3	1107	1	8
	420	300	29	120
	602	600	0	2
	350	346	1	4
	969	943	3	26
	1139	1131	1	8
	771	770	0	1
	800	520	35	280
Average	780.7	732.3	7	48
SD	375.3	358.4	10	71
Median	710	616	5	31
IQR 1	545	520.8	1	6.6
IQR 3	916.5	870.3	7	58

*All specimen weights recorded intraoperatively and in the pathology laboratory are shown. Actual and percentage decreases of specimen weight are calculated. Specimen weight differences and percentages with a (+) in front indicate an increase in recorded weight in the pathology laboratory from the intraoperative weight. Average and median values for each data column are demonstrated at the bottom of the table. The interquartile range for each data column is shown, demonstrating the 25th percentile (IQR 1) and 75th percentile (IQR 3).

**Table 3:** University of Louisville Hospital insurance reimbursement^*^

	$ First side	$ Second side	Weight requirement
**Anthem**	**1167.99**	**584.00**	**Schnur scale**
**Humana**	**877.09**	**438.55**	**750 g each**
**Indiana Medicaid**	**766.21**	**766.21**	**750 g each**
**Ky Medicaid**	**622.36**	**622.36**	**750 g each**
**Medicare**	**615.05**	**615.05**	**Insurance scale**
**Passport**	**639.38**	**639.38**	**750 g each**
**Tricare**	**869.33**	**434.66**	**Insurance scale**
**United Healthcare**	**1635.01**	**817.50**	**750 g each**

*Third-party payment data of accepted third-party payers at University of Louisville Hospital demonstrate actual reimbursement and weight requirement policy of each health insurance provider.
